# *Blattella germanica* Selects Microbiota Taxa from Feces and Environmental Inputs

**DOI:** 10.3390/insects17060615

**Published:** 2026-06-10

**Authors:** Samuel Piquer-Esteban, Vicente Pérez-Brocal, Rebeca Domínguez-Santos, Amparo Latorre, Carlos García-Ferris, Andrés Moya

**Affiliations:** 1 Institute for Integrative Systems Biology (I2SysBio), University of Valencia and Spanish Research Council, 46980 Paterna, Spainrebeca.dominguez@uv.es (R.D.-S.); amparo.latorre@uv.es (A.L.); carlos.garcia.ferris@uv.es (C.G.-F.); 2 Genomic and Health Area, Foundation for the Promotion of Sanitary and Biomedical Research of the Valencia Region (FISABIO), 46020 Valencia, Spain; 3 Department of Biochemistry and Molecular Biology, University of Valencia, 46100 Burjassot, Spain

**Keywords:** cockroach, microbiota acquisition, environmental influence, coprophagy, fecal transplantation, host effect

## Abstract

Cockroaches display a double symbiosis: an obligate intracellular one with *Blattabacterium* spp., and a complex extracellular non-vertically transmitted gut microbiota, that may be affected by horizontally transmitted factors. Four experiments using 16S rRNA gene amplicon sequencing were conducted to analyze the microbiota of the hindgut and feces of adult cockroaches. They aimed to understand the influence of the environment and feces on the acquisition and development of the hindgut microbiota. We observed that sample type (hindgut vs. feces), rearing conditions (environment, i.e., place and diet), coprophagy, and host influenced microbiota composition. Cockroaches initially germ-free, placed in non-sterile conditions and with blocked parental coprophagy, were unable to develop the normal microbiota of the control population, demonstrating that coprophagy is essential for acquiring a normal microbiota. This also showed that, in the absence of parental fecal input, the cockroach gut microbiota is strongly diminished. Moreover, when exploring fecal microbiota differences among three cockroach species, the greatest divergence was observed between *Periplaneta americana* and *Blattella germanica*, with *Blatta orientalis* occupying an intermediate position. Therefore, *P. americana* was selected for fecal transplantation on *B. germanica*. This transplantation experiment indicates that different species select different gut microbes, and that even when they receive feces from other species, only some of those bacteria are retained. Overall, these results suggest that beyond other factors, the host species had the strongest influence on shaping the cockroach gut microbiota.

## Simple Summary

Cockroaches harbor a rich and complex bacterial community that provides them with nutritional benefits and enables them to adapt to their environment. In this study, we explored how adult cockroaches acquire the bacteria that form their gut community. To do this, we performed four experiments to assess the roles of the environment, the bacteria present in feces, and the cockroach host species. We found that factors such as diet and the location where insects are raised influence the bacteria they acquire. Feces intake is also essential for the development of the gut microbiota of young cockroaches by coprophagy. However, the most notable influence came from the host species, which can select which bacteria are kept in its gut. Even when a cockroach received feces from another species, it retained only a fraction of those bacteria. These results indicate that, beyond the influence of the environment and feces, the host species plays the biggest role in shaping and maintaining its gut bacterial community, helping explain why each species has its own characteristic microbiota.

## 1. Introduction

Insects are the most diverse and abundant metazoans on Earth, with around one million described species [1]. Most of them are involved in symbiotic associations with bacteria, ranging from mutualistic to parasitic. These interactions can have significant effects on the reproduction and metabolism of the host insects [2], among other aspects of the host’s life. In addition to these well-known effects, insect-associated bacteria also contribute to nutrient metabolism, immune regulation, and ecological adaptation, underscoring their evolutionary relevance [3,4]. Broadly speaking, two types of symbiosis occur between insects and bacteria: intracellular symbiosis, in which bacteria reside within specialized host cells and typically supply essential nutrients absent from the host’s unbalanced diet [5], and extracellular symbiosis, such as that involving the gut microbiota, where symbionts play different roles in the host’s physiology like digestion, detoxification, immune modulation, and resilience to environmental fluctuations, allowing both to thrive in constantly changing environments [6]. These two systems include functionally diverse associations in insects, which can combine long-term obligate endosymbionts with highly complex and environment-dependent gut microbiotas. This combined presence of intracellular and gut-associated symbioses provides a unique framework for exploring host–microbe interactions in insects. In urban environments, where several pest insects are of public-health concern, understanding host–microbiota interactions is also relevant for pest and disease management.

Among these urban pest insects, cockroaches are especially interesting because they harbor both symbiotic systems: the obligate intracellular endosymbiont *Blattabacterium* spp. (hereinafter, *Blattabacterium*) a vertically transmitted Gram-negative bacterium essential for the host’s survival and involved in nitrogen metabolism [7,8,9,10,11,12]. They also harbor a complex and rich gut microbiota, which has been characterized for its composition, assembly during development, and putative roles in host physiology [10,13,14,15,16,17,18,19,20,21,22,23]. Beyond nutritional functions, the cockroach gut microbiota may also influence host immunity and interactions with pathogens. Studies in the German cockroach *Blattella germanica* have shown that gut bacteria can modulate susceptibility to entomopathogenic fungi, revealing microbiota-mediated effects on host–pathogen interactions [24].

In insects, gut diversity can be affected by many factors, both environmental and host genetics. For example, the strong correlation between host species and gut microbiota composition in omnivorous gregarious cockroaches suggests that host species background plays a key role in shaping microbiota composition [22]. Other factors, like habitat, season, life stage, sex, treatment (antibiotics, pesticides, etc.), and diet, also affect gut microbiota composition [15,19,25,26]. However, despite extensive research, the relative contributions of host and environmental exposure remain unresolved. This “host genetics vs. environment” question is particularly relevant in cockroaches, where both strong social behaviors and environmental microbial sources shape the acquisition of the microbiota. Clarifying this issue may help elucidate the stability, evolution, and plasticity of insect–microbe associations.

*B. germanica*, a social insect from the order Blattodea, exhibits group behaviors, including selection of populated shelters, kin recognition, and chemical communication [27,28,29,30]. Sharing shelter promotes coprophagy, where juveniles feed on adult feces, which is involved in gut microbiota establishment and maturation [31,32]. *Blattabacterium* is the only bacterium present in the ootheca, confirming the absence of vertical transmission of gut microbiota [10]. Subsequently, newborn nymphs are germ-free and acquire bacteria in their first nymphal instar. By the second nymphal stage, their gut microbiota is established, primarily through feeding on adult feces via coprophagy [19], an indirect form of vertical transmission mediated by social behavior and distinct from the transovarial transmission of *Blattabacterium*. Our recently developed gnotobiotic (germ-free) *B. germanica* population, lacking microbiota but with *Blattabacterium* present [33], enables the study of gut microbiota differences through different approaches, providing a model to understand the roles of the host, gut tissue, environment, and other factors in microbiota acquisition. This system allows these influences to be examined under controlled and complementary experimental conditions.

This study aimed to better understand the influence of the environment, feces, and host species on the acquisition and development of the gut microbiota in adult cockroaches. Four experiments were conducted to (i) compare the microbiota of two *B. germanica* populations with the same genetic background but separated for two decades, (ii) study the environmental impact in one generation on germ-free coprophagy-blocked *B. germanica* populations placed in non-sterile conditions, (iii) compare the fecal microbiota of *B. germanica*, *Periplaneta americana*, and *Blatta orientalis*, and (iv) elucidate the role of the host in gut microbe selection following interspecies fecal transplantation.

## 2. Materials and Methods

### 2.1. Rearing Conditions of Cockroaches

All cockroach colonies were maintained in climatic chambers in three locations: at the Institute for Integrative Systems Biology (I2SysBio, Valencia, Spain, geographic coordinates 39.516713, −0.423122) (Inkoa, Erandio, Spain), at the Cavanilles Institute of Biodiversity and Evolutionary Biology (ICBiBE, Valencia, Spain, geographic coordinates 39.514648, −0.425886) (Ibercex, Madrid, Spain), and at the Institute of Evolutionary Biology (IBE, Barcelona, Spain, geographical coordinates 41.3851913, 2.1960337) (Sanyo MLR-351, Moriguchi, Japan). Insects were reared at 25 °C, 60% humidity, and a photoperiod of 12L:12D. They were fed with three different dog food pellets—SAFE 125 (Rettenmaier Ibérica, Barcelona, Spain) and Teklad Global diet 2021C (Envigo, Indianapolis, IN, USA), both with a 21% protein content, and Vitality (Elmubas Ibérica, Gipuzkoa, Spain)—depending on experimental design and on the rearing location (Supplementary Table S1), and water was provided ad libitum.

### 2.2. Obtaining Germ-Free Cockroaches and Quality Control

Mature oothecae were collected from synchronized *B. germanica* adults, and their surfaces were sterilized by immersion for 20 s in 0.1% SDS, followed by 12 min of 2% peracetic acid with gentle shaking. Subsequently, the oothecae were rinsed twice with sterile laboratory-grade purified type II water (conductivity 0.067–1.0 µS/cm, resistance 15–10 MΩ·cm, retention rate for bacteria and particles 99%), obtained using the commercial purification system Puranity TU 12+ (VWR, Leuven, Belgium), to remove the antiseptic solution and transferred to sterile gauze to remove excess water. The aseptic oothecae were incubated individually in sterile 50 mL tubes at 25 °C until hatching, as previously described [33].

After hatching, quality control tests were conducted to assess the efficacy of the sterilization. Two nymphs from each ootheca and the ootheca itself were tested for microbial growth by plating them on BHI agar plates, which were incubated at 37 °C for 24 h and then transferred to 25 °C for two weeks. In the event of microbial growth detection, contaminated batches were discarded. The protocol for verifying sterility was established according to our previously described and validated methodology [33].

### 2.3. Insect Dissection

Female cockroaches of *B. germanica* were anesthetized with CO_2_ and maintained on ice until dissection. The insects were cleaned with 10% bleach and 70% ethanol solution, followed by a double wash with laboratory-grade purified type II water. Subsequently, they were placed in a supine position on a silicone plate and fixed with entomological pins. The hindgut tissue was collected, opened longitudinally, and cleaned with Krebs–Ringer bicarbonate buffer (Sigma-Aldrich, Burlington, MA, USA). Finally, the hindgut tissue was immediately frozen with liquid nitrogen and stored at −80 °C.

Given the technical constraints associated with generating and maintaining germ-free individuals, together with the stringent sterility controls that required discarding any contaminated oothecae, sample sizes per group were necessarily limited. Nevertheless, this design ensured consistent recovery of high-quality hindgut material for downstream microbiota analyses. Fecal sample numbers were adjusted accordingly to maintain a balanced design across sample types.

### 2.4. Experimental Designs

**Figure 1:**
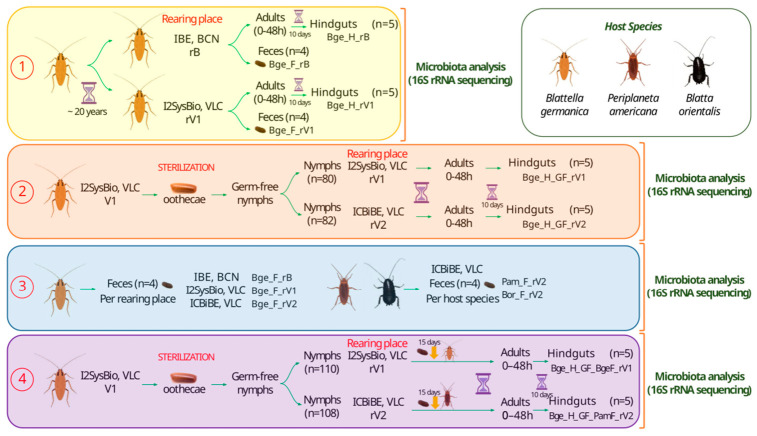
Experimental designs. The four experiments include the cockroach species used, the rearing conditions, and the sample types. IBE, BCN: Institute of Evolutionary Biology, Barcelona; I2SysBio, VLC: Institute for Integrative Systems Biology, Valencia; ICBiBE, VLC: Cavanilles Institute of Biodiversity and Evolutionary Biology, Valencia; rB: rearing place Barcelona (IBE); rV1: rearing place Valencia1 (I2SysBio); rV2: rearing place Valencia2 (ICBiBE); H: hindgut; F: feces; GF: initially germ-free individuals; Bge: *Blattella germanica*; Pam: *Periplaneta americana*; Bor: *Blatta orientalis*; BgeF: fecal input from *B. germanica* during transplantation; PamF: fecal input from *P. americana* during transplantation.

A graphical summary of the experimental design for the four experiments conducted in this study, including the cockroach species, the rearing locations, and the number and type of samples used, is shown in Figure 1.

*Experiment 1*. To investigate the contribution of the host and the environmental effects on *B. germanica*, two laboratory populations with the same genetic background but separated for nearly two decades were studied. The original population (rB) was reared at the IBE by Dr. Xavier Bellés’ group at 29 °C, but was adjusted for one generation at 25 °C for this experiment. The second population (rV1) was established at the I2SysBio from the initial population in the year 2000 and has been raised at 25 °C since then. Both populations were fed dog food pellets—SAFE 125 and Teklad Global diet 2021C, respectively. For this study, in 2019, adults were collected from a synchronized population at 0–48 h post-ecdysis. Five females from each population were dissected 10 days after the population started, and their hindgut tissues were collected. Additionally, four fecal samples were collected from adults of each population.

*Experiment 2*. To investigate the impact of different environments (with the same diet and excluding feces) on hindgut microbiota, we initiated the study following our previously published protocol [31] to obtain germ-free nymphs, which were subsequently placed in separate environments. To do that, mature oothecae were collected from females in a synchronized, sex-balanced cockroach population established at I2SysBio. The oothecae were sterilized following the protocol described previously. Once hatching had occurred and sterility had been confirmed, the nymphs emerged from the sterilized ootheca. They were used to initiate two populations maintained in separate environments under non-sterile conditions, without parental feces, to prevent coprophagy and interrupt fecal transmission of the microbiota. One population, comprising 80 nymphs, was placed in a climatic chamber at I2SysBio (rV1), while the other population, comprising 82 nymphs, was placed in a climatic chamber at ICBiBE (rV2). Both populations were fed with the same dog food pellets (Teklad Global diet 2021C). Adults were separated within 48 h after ecdysis and maintained for 10 days. Hindgut tissues from five females in each population were then dissected.

*Experiment 3*. Feces from three laboratory-reared species were obtained to compare the fecal microbiota among cockroach species. Each location had its own dog food pellets for feeding. Specifically, three populations of *B. germanica* raised in climatic chambers at rB, rV1, and rV2, plus one population of *P. americana* and another of *B. orientalis*, both raised at rV2, were examined. Four fecal samples per population were analyzed.

*Experiment 4*. To investigate how the host impacts the gut microbiota, germ-free populations of *B. germanica* were generated, as previously described. Following hatching and verification of axenic status, germ-free nymphs were divided into two populations. One group of 110 nymphs was given *B. germanica* feces for 15 days and reared in a climatic chamber at rV1. The other population of 108 nymphs was provided with *P. americana* feces for 15 days and reared at rV2. Both populations were fed with the same dog food pellet (Teklad Global diet 2021C). Adults were collected within 48 h after ecdysis and maintained for 10 days. Each transplanted population was maintained in the environment corresponding to the feces donor species to prevent cross-contamination in the rearing chamber with the donor species’ microbiota, especially to avoid cross-contamination of the control population maintained in rV1 with *P. americana* microbiota. Hindgut tissues from five females in each group were then dissected and analyzed.

In all experiments, individuals within each treatment group were collected from the same colony box maintained under controlled laboratory conditions. For this reason, the effective experimental unit is the colony rather than the individual insect, and the analyzed samples should be considered subsamples rather than fully independent biological replicates. Establishing multiple independent colony boxes per treatment was not feasible due to technical constraints in generating and maintaining germ-free cockroach populations, as well as the strict sterility requirements of the experimental setup. All samples were processed independently for DNA extraction, sequencing, and downstream analyses.

### 2.5. DNA Extraction and Metabarcoding Sequencing

The total DNA of hindgut and fecal samples was extracted using the Jet-Flex Genomic DNA Extraction Kit (Genomed, Leinfelden-Echterdingen, Germany) with modifications [20]. Metabarcoding sequencing was conducted at the facilities of FISABIO, Valencia (Spain). The extracted DNA served as a template for amplification using specific primers of the V3–V4 variable region of the 16S rRNA gene. The resulting amplicons were sequenced as 2 × 300 bp paired-end reads on a MiSeq platform (Illumina, San Diego, CA, USA).

### 2.6. Sequence Processing and Taxonomic Assignment

Primer removal was carried out on raw sequence files using Cutadapt v3.2 [34], and untrimmed sequences were discarded. Reads were processed using DADA2 v1.18.0 [35]. Quality trimming and filtering were performed for forward (260 bp, 3 maxEE) and reverse (210 bp, 5 maxEE) reads. Amplicon sequence variants (ASVs) were determined, paired-end reads were merged, and chimeric sequences were eliminated. Taxonomy assignment was performed using IDTAXA classifier [36] from DECIPHER v2.18.0 [37] against SILVA v138 reference taxonomy database [38] using a 50% threshold. Unclassified ASVs, those identified as mitochondria or chloroplasts, and those not belonging to the Bacteria or Archaea domains, or matching the *Blattabacterium* endosymbiont, were filtered. For taxonomic classification at the genus level, ASV counts were grouped by the last identifiable taxonomic level, with higher levels designated as unclassified.

### 2.7. Microbiome Analysis

The analyses were carried out primarily in R v4.6.0, using Microbiome v1.22 [39], Phyloseq v1.44.0 [40], and Vegan v2.6-4 [41]. A significance threshold of 0.05 was set for all analyses.

For alpha diversity analysis, indexes were computed at the ASV level using the Microbiome package. Pairwise comparisons studied differences between groups. After evaluating normality with the Shapiro test and homogeneity of variance with the Levene test, we used the parametric *t*-test with equal variances for Observed Richness and the unequal-variance *t*-test for Shannon’s Diversity Index (Supplementary Table S2). All *p*-values were adjusted by the Benjamini–Hochberg (BH) method.

For beta diversity analysis, Aitchison distances (Euclidean distance with CLR transformation) were used. Abundance data were centered log-ratio-transformed (CLR) using the Microbiome package. Only taxa with abundance above 0.01% in at least one sample were considered. Inter-sample differences were investigated employing a Principal Component Analysis (PCA). Differences between groups were tested with a permutational multivariate analysis of variance (PERMANOVA) using 10,000 permutations with Vegan’s “adonis2” function at the ASV (Supplementary Table S3) and genus (Supplementary Table S4) levels. Pairwise comparisons between groups were corrected using the BH method. A model using the “betadisper” function was also calculated to test group homogeneity later using the “permutest” function. Moreover, pairwise homogeneity differences between groups were also analyzed using TukeyHSD on the betadisper objects. Finally, variables of interest were analyzed using a PERMANOVA at the genus level for each experimental design with different models (Supplementary Table S5). Furthermore, for each experimental-design PCA, the “scores” function from the Vegan package was used to obtain the ordination scores of the top 50 taxa for the first two principal components. Significant differences in taxon abundances were evaluated using ALDEx2 v1.32.0 [42]. Only taxa with abundance above 0.01% in at least one sample were considered. GLM and KW tests for one-way ANOVA of two or more conditions were used, using 1000 Monte Carlo samples. All *p*-values were adjusted using the BH method. Only significant taxa in both tests were conserved. Heatmaps based on Z-scored average relative abundances were generated using ComplexHeatmap v2.16.0 [43].

Taxa similarities between groups of interest were analyzed using ComplexUpset v1.3.3 [44] and ggvenn v0.1.10 [45] at the genus level. For the resulting plots, a taxon was considered to be present in the groups if it was detected in at least one sample.

Core microbiome analysis was performed at the genus level using the Microbiome package, with relative abundance and prevalence thresholds of 0.0001 and 1 (present in all samples), respectively. Taxonomy graphics were generated using Metacoder v0.3.6 [46].

## 3. Results

Sample codes follow a unified structure identifying the host species (Bge: *Blattella germanica*; Pam: *Periplaneta americana*; Bor: *Blatta orientalis*), sample type (H: hindgut; F: feces), and the experimental conditions, including: germ-free status (GF: initially germ-free individuals), fecal donor during transplantation (BgeF: fecal input from *B. germanica*; PamF: fecal input from *P. americana*), and rearing environment (rB: rearing place Barcelona in IBE; rV1: rearing place Valencia1 in I2SysBio; rV2: rearing place Valencia2 in ICBiBE). For example, “Bge_H_GF_PamF_rV2” corresponds to a *B. germanica* hindgut sample obtained from a germ-free recipient transplanted with *P. americana* feces and reared in environment V2.

### 3.1. Global Microbial Diversity

Across all experiments (Figure 1), 50 samples were analyzed (Supplementary Table S1). After processing and filtering, 6,146,692 reads were retained, comprising 3004 ASVs with an average of 122,934 reads per sample (minimum: 34,810 and maximum: 1,041,372 reads).

The hindgut and fecal microbiotas of cockroaches were complex and diverse (Supplementary Figure S1). With 19 phyla identified, Bacteroidota was usually the most abundant (43.09% on average), followed by Firmicutes (24.68%), Proteobacteria (12.55%), Fusobacteriota (9.18%), and Desulfobacterota (5.45%). At the genus level, most of the community remained unclassified (60.18% on average). Among classified genera, *Fusobacterium* was the most abundant (9.18%), followed by *Dysgonomonas* (8.58%) and *Desulfovibrio* (3.96%), all of which were highly depleted in coprophagy-blocked samples.

**Figure 2:**
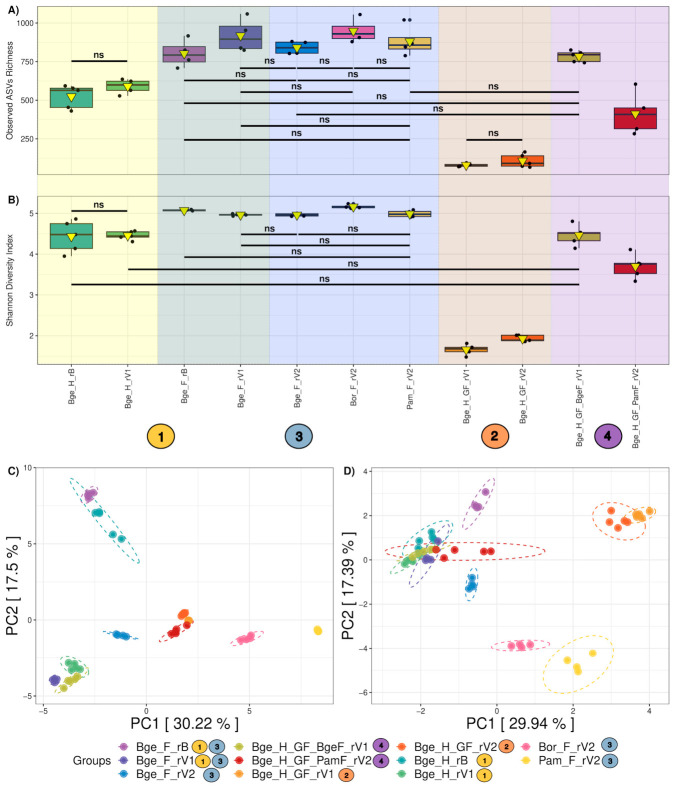
Global diversity analysis. Alpha diversity metrics, including Observed Richness (**A**) and Shannon Diversity Index (**B**), were obtained at the ASV level for the different groups. In the box plots, the black line within the box marks the median, and the yellow triangle is the mean. Background colors and identifying labels match those used to name the experiments in Figure 1. Not significant (ns) comparisons in pairwise *t* tests (adjusted *p*-value > 0.05) are indicated, while the rest of the possible comparisons were significant. Beta diversity was also explored using a PCA of CLR-transformed data at the ASV (**C**) and genus (**D**) levels. Ellipses represent 95% confidence intervals. For further details, see interactive PCAs in Supplementary Figures S2 and S3. Experiment labels are shown next to the legend for each group. Sample nomenclature follows the structure described at the beginning of the Results section. Circles with the color and number of the experiment described in Figure allow each of the groups to be related to the specific experiment in which it has been used.

For alpha diversity (Figure 2A,B), fecal samples exhibited the highest richness and diversity, with significant differences observed (Supplementary Table S2). Among feces, *B. orientalis* displayed higher richness and diversity than *B. germanica* and *P. americana*. As expected, coprophagy-blocked hindgut samples exhibited the lowest richness and diversity (Bge_H_GF_rV1 and Bge_H_GF_rV2) with significant differences when compared to other groups. The standard *B. germanica* hindgut samples (Bge_H_rB and Bge_H_rV1) showed intermediate values between those from fecal samples and those from coprophagy-blocked hindgut samples, with no significant differences between the two groups. Finally, regarding hindgut transplanted samples, those transplanted with *P. americana* feces (Bge_H_GF_PamF rV2) displayed significantly lower richness and diversity than *B. germanica* intra-species feces transplants (Bge_H_GF_BgeF rV1), which was also the case when comparing with control *B. germanica* hindgut samples. Meanwhile, the *B. germanica* intra-species fecal transplants showed no significant differences in diversity compared with control *B. germanica* hindgut samples, but were significantly higher in richness.

When studying beta diversity at ASV and genus levels (Figure 2C,D), great heterogeneity was shown, with a variation in the PCA explained by the first axes of 47.72% and 47.33%, which increased to 59.02% and 59.46% when adding the third component (Supplementary Figures S2 and S3), respectively. A noticeable effect of the host and the cockroach species of origin of the microbiota was observed, and, regarding *B. germanica*, coprophagy-blocked hindguts (Bge_H_GF_rV1 and Bge_H_GF_rV2) showed greater differences from the rest of the samples. Differences by rearing place were also found (rB, rV1, and rV2). Interestingly, hindgut transplanted with *P. americana* feces (Bge_H_GF_PamF_rV2) had an intermediate position at the ASV level. In contrast, at the genus level, it turned out to be more closely related to the rest of the *B. germanica* samples. These differences were significant across all groups when using a PERMANOVA at both taxonomic levels. Furthermore, permutest (*p* = 0.0001 at the ASV level, and *p* = 0.0156 at the genus level) and subsequent TukeyHSD revealed the presence of variance differences between groups (25 pairs at the ASV level and 2 pairs at the genus level), indicating that dispersion differences contribute to the observed multivariate differences in community composition (Supplementary Tables S3 and S4).

### 3.2. Comparison of the Gut Microbiota of Twinned Populations of B. germanica

**Figure 3:**
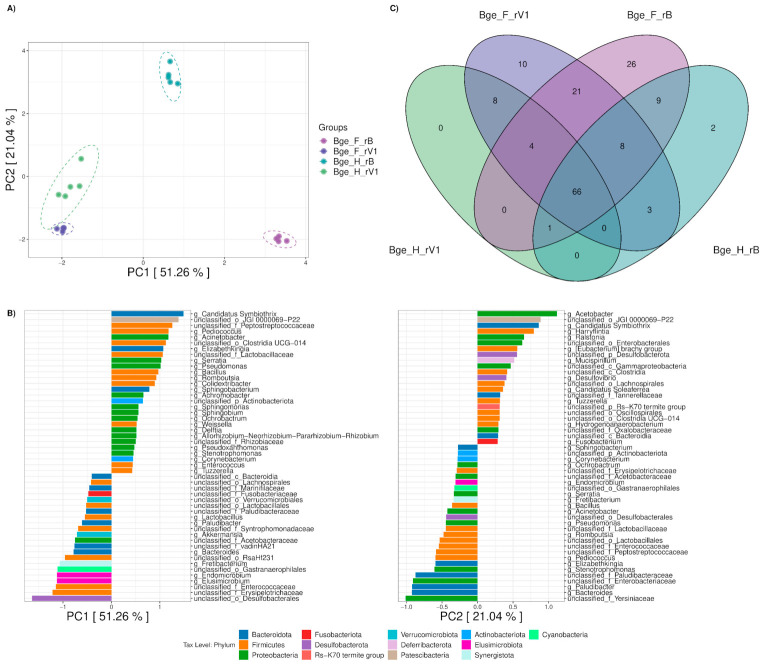
Experiment 1: Comparing twinned populations of *B. germanica*. Beta diversity was explored using a PCA of CLR-transformed genus-level data (**A**). Ellipses represent 95% confidence intervals. The ordination scores of the top 50 taxa are shown for the first two principal components, PC1 and PC2 (**B**). A Venn diagram of the intersections between genera from the different experimental groups is also shown (**C**). Bge_F_rB: Barcelona feces; Bge_H_rB: Barcelona hindgut; Bge_F_rV1: Valencia1 feces; Bge_H_rV1: Valencia1 hindgut.

The first experiment aimed to compare two populations of *B. germanica* with the same genetic background, which had remained separated for 20 years, reared in different environmental conditions (rB and rV1) and fed different diets (both with 21% protein content). When comparing feces and hindgut microbiotas at the genus level (Figure 3A,B), the first component separated samples based on rearing place. While examining their associated taxa, unclassified Desulfobacterales, *Elusimicrobium*, and *Endomicrobium* characterized rV1, while others, such as Candidatus *Symbiothrix*, *Pediococcus*, and *Acinetobacter*, drove differences toward rB. Meanwhile, the second component mainly separated samples by sample type (feces vs. hindgut). Unclassified Yersiniaceae, *Bacteroides*, and *Paludibacter* characterized fecal samples, while others, such as *Acetobacter*, *Harryflintia*, and *Ralstonia*, drove differences toward hindgut samples. Different PERMANOVA models were performed (Supplementary Table S5-A1). In all models, rearing place explained most of the between-sample variation (R^2^ = 0.446), followed by sample type (R^2^ = 0.195), both of which were significant. Interaction effects between the two variables were also detected, easily explained by the greater intra-similarity among rV1 samples.

Despite differences, most taxa were present in all four groups (Figure 3C and Supplementary Table S6-A1). Furthermore, 21 genera were found exclusively in fecal samples from both locations. More genera were identified exclusively in feces from rB compared to rV1. Additionally, unique genera in both feces and hindgut samples from each location were identified.

### 3.3. Exploring the Environmental Effect (Excluding Diet) and Coprophagy on the Gut Microbiota

**Figure 4:**
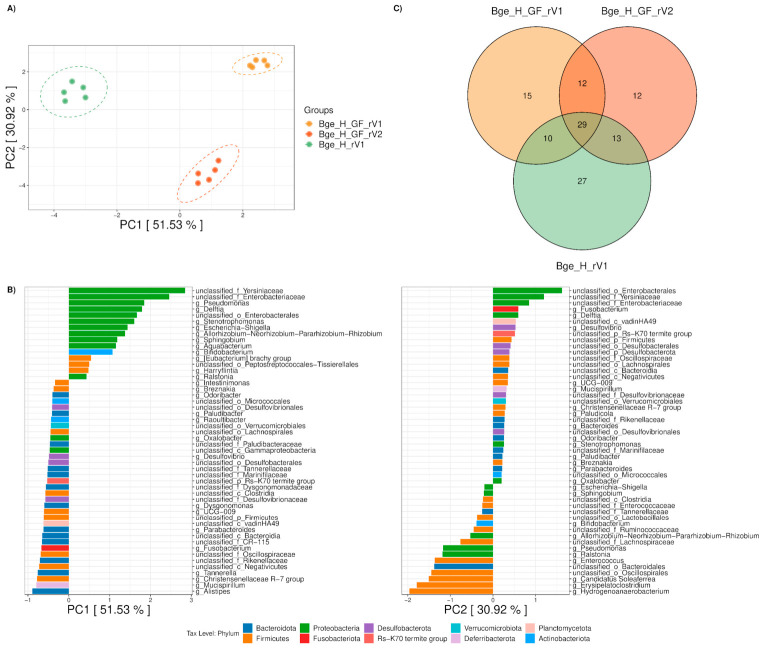
Experiment 2: Environmental effects on coprophagy-blocked *B. germanica* cockroaches. Beta diversity was explored using a PCA of CLR-transformed genus-level data (**A**). Ellipses represent 95% confidence intervals. The ordination scores of the top 50 taxa are shown for the first two principal components, PC1 and PC2 (**B**). A Venn diagram of the intersections between genera from the different experimental groups is also shown (**C**). Bge_H_rV1: Valencia1 hindgut (control condition); Bge_H_GF_rV1 and Bge_H_GF_rV2: Valencia1 and Valencia2 hindguts in parental coprophagy-blocked condition, respectively.

The second experiment studied the effects that the environment and coprophagy exert on the gut microbiota. For this purpose, oothecae of *B. germanica* from the same rearing place (rV1) were subjected to a sterilization process, and germ-free nymphs were placed in two different non-sterile environments (rearing places rV1 and rV2) with the same diet, avoiding contact with parental feces. When comparing hindgut microbiota from control individuals (Bge_H_rV1) and cockroaches with blocked parental coprophagy reared in environments rV1 and rV2 (Bge_H_GF_rV1 and Bge_H_GF_rV2), the first component separated samples based on the presence or absence of parental coprophagy (Figure 4A,B). Upon examining their associated genera, unclassified Enterobacterales, *Pseudomonas*, and *Delftia* showed differences toward hindgut samples from individuals without parental coprophagy. In contrast, others, such as *Alistipes*, *Mucispirillum*, and *Tannerella*, were more closely related to the control hindgut samples from cockroaches with parental coprophagy. Meanwhile, the second component separated samples by rearing place. Taxa like unclassified Enterobacterales drove differences towards rV1, while others, such as *Hydrogenoanaerobacterium* and *Erysipelatoclostridium*, characterized rV2. Different PERMANOVA models were performed (Supplementary Table S5-A2). In all models, the absence of parental coprophagy through initial germ-free conditions explained most of the between-sample variation (R^2^ = 0.496), followed by rearing place (R^2^ = 0.317), both of which were significant.

Despite differences, 29 taxa were present in all three groups (Figure 4C and Supplementary Table S6-A2). However, the majority are highly depleted in coprophagy-blocked individuals (Bge_H_GF_rV1 and Bge_H_GF_rV2), except for a few taxa that became highly abundant, displacing the rest of the microbial community (Supplementary Figure S1B). Additionally, 27 taxa were found exclusively in rV1′s hindgut control. These taxa were also present in rV1 and rV2 *B. germanica* feces (Supplementary Table S6), but were absent in hindguts from coprophagy-blocked individuals, indicating that they are acquired from feces and not from the environment.

### 3.4. Comparison of the Fecal Microbiota of Three Cockroach Species

**Figure 5:**
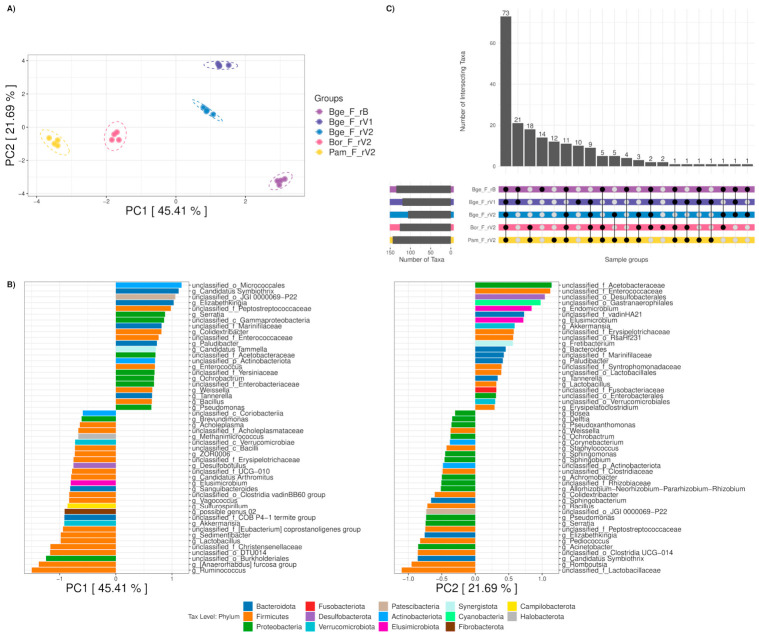
Experiment 3: Exploring the feces microbiota of different species of cockroaches. Beta diversity was examined using a PCA of CLR-transformed genus-level data (**A**). Ellipses represent 95% confidence intervals. The ordination scores of the top 50 taxa are shown for the first two principal components, PC1 and PC2 (**B**). An upset plot of the intersections between genera from the different experimental groups is also shown (**C**). Bge_F_rB: *B. germanica* in Barcelona; Bge_F_rV1: *B. germanica* in Valencia1; Bge_F_rV2: *B. germanica* in Valencia2; Bor_F_rV2: *B. orientalis* in Valencia2; Pam_F_rV2: *P. americana* in Valencia2.

The third experiment aimed to compare the fecal microbiota of *B. germanica* and two other cockroach species to identify the most suitable donor for subsequent inter-species fecal transplantation into germ-free *B. germanica*. When comparing the feces of the three cockroach species at the genus level (Figure 5A,B), the first component separated samples based on host species. The fecal microbiota of *P. americana* was the most divergent from *B. germanica*, while *B. orientalis* occupied an intermediate position. Upon examining their associated genera, taxa such as unclassified Micrococcales, Candidatus *Symbiothrix*, *Elizabethkingia*, or *Serratia* characterized *B. germanica* feces. In contrast, others, such as *Ruminococcus* and unclassified Burkholderiales, drove differences toward *B. orientalis* and *P. americana*. Meanwhile, the second component separated the samples by environment (i.e., rearing place, each with its associated diet). Samples in rV1 were arranged at the top, while rV2 and rB were at the center and bottom, respectively. Taxa like unclassified Acetobacteraceae and unclassified Enterococcaceae drove differences towards the positive axis, while others, such as Candidatus *Symbiothrix*, moved it towards the negative axis. Different PERMANOVA models were performed (Supplementary Table S5-A3). In all models, the host always explained most of the between-sample variation (R^2^ = 0.367–0.56), followed by the rearing place (R^2^ = 0.343), both being significant.

Despite the observed differences, 73 genera were shared by the feces of the three cockroaches (Figure 5C and Supplementary Table S6-A3). Only one taxon was unique to *B. germanica* and present in its three groups, while two genera were unique to *B. orientalis*. In contrast, 12 genera were exclusively found in *P. americana*. Furthermore, 18 genera were shared by *B. orientalis* and *P. americana*, while absent in *B. germanica*. Moreover, all *B. germanica* populations shared two genera with *B. orientalis* but were absent from *P. americana*. Therefore, *P. americana* was chosen as the fecal donor for the cross-species fecal transplantation experiment, as it displayed the most differences and taxa of its own with respect to the recipient *B. germanica*.

### 3.5. Inter-Species Cockroaches’ Fecal Transplantation

**Figure 6:**
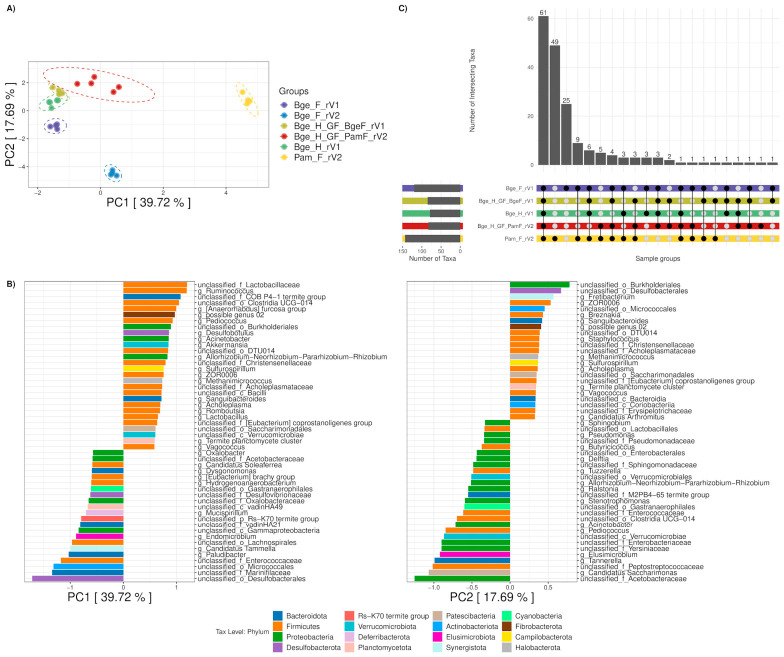
Experiment 4: Inter-species cockroaches’ fecal transplantation. Beta diversity was explored using a PCA of CLR-transformed genus-level data (**A**). Ellipses represent 95% confidence intervals. The ordination scores of the top 50 taxa are shown for the first two principal components, PC1 and PC2 (**B**). Bge_F_rV2 group (*B. germanica* feces in Valencia2) was included only in the PCA analysis to differentiate possible environmental effects better. An upset plot of the intersections between genera from the different experimental groups is also shown (**C**). Bge_H_rV1: *B. germanica* hindgut in Valencia1 (hindgut control); Bge_F_rV1: *B. germanica* feces in Valencia1 (intra-species feces); Pam_F_rV2: *P. americana* feces in Valencia2 (inter-species feces); Bge_H_GF_BgeF_rV1: hindgut of germ-free transplanted *B. germanica* in Valencia1 (intra-species transplant); Bge_H_GF_PamF_rV2: hindgut of germ-free transplanted *B. germanica* in Valencia2 with *P. americana* feces (inter-species transplant).

The fourth experiment explored the effect of the host on the microbiota acquisition process by using fecal transplantation between cockroach species. For this purpose, oothecae of *B. germanica* from the colony reared at rV1 were subjected to a sterilization process. The nymphs were provided with different input feces from *B. germanica* (placed in rV1) and *P. americana* (placed in rV2 to avoid cross-contamination of the *B. germanica* population at rV1 with *P. americana* feces) and reared with the same diet. Microbiota of feces (Bge_F_rV1 and Pam_F_rV2) and the host’s control hindgut (Bge_H_rV1) were compared with the hindgut microbiota of transplant recipients (Figure 6A,B). Host species mainly influenced the first component, although rearing place also played a role. Taxa such as *Ruminococcus* or unclassified Lactobacillaceae drove differences toward the positive axis for *P. americana* and rV2. In contrast, others, such as unclassified Marinifilaceae and unclassified Desulfobacterales, drove differences toward the negative axis related to *B. germanica* and rV1. Meanwhile, the second component appeared to reflect a combination of factors, including sample type (hindgut and feces), transplant, and input feces species. Different PERMANOVA models were performed (Supplementary Table S5-A4). In all models, the host always explained more of the variation between samples than other variables (R^2^ = 0.146–0.362). All variables were significant in all models. 

In total, 61 taxa were shared across all groups (Figure 6C and Supplementary Table S6-A4). The majority of taxa exclusive to feces were restricted to either *P. americana* or *B. germanica*, with 49 and 25 genera, respectively. Only nine genera were shared by the feces of both species and absent in hindguts (*Akkermansia*, *Bacillus*, *Corynebacterium*, *Dialister*, *Glutamicibacter*, *Lactobacillus*, *Romboutsia*, unclassified Peptostreptococcaceae, and unclassified_o_Clostridia UCG-014). Taxa potentially influenced by the germ-free transplant process were also identified, with three taxa present in the control *B. germanica* hindgut and feces but absent in transplanted hindguts. Additionally, four taxa were detected in all groups except the control *B. germanica* hindgut. Likewise, some taxa related to feces were found, with five taxa exclusive to *P. americana* and one exclusive to *B. germanica*.

A differential abundance analysis was also performed at the genus level (Supplementary Figure S4 and Table S7). A total of 93 taxa showed significant differential abundance. The group of hindguts transplanted with *B. germanica* feces and the control hindgut of *B. germanica* clustered together. The closest group to these was the feces of *B. germanica*. These three groups presented both particular and different taxa clusters, which were generally more enriched in them. The next group in clustering was the hindgut transplanted with *P. americana* feces. It mainly presented particular enriched groups, and others shared with the furthest group, which consisted of feces of *P. americana*. The latter had the highest number of enriched taxa among the groups.

### 3.6. Analysis of the Core Microbiome

Only taxa detected in samples under control conditions were considered for core analyses to explore highly prevalent taxa in the hindgut of *B. germanica* and feces of the three cockroach species (Supplementary Figure S5 and Table S8). Genera like *Fusobacterium*, *Dysgonomonas*, *Alistipes*, Candidatus *Soleaferrea*, and *Desulfovibrio* were shared across all cores. Generally, they exhibited high abundances, whereas other common to all core taxa, including *Oxalobacter*, *Robinsoniella*, *Hydrogenoanaerobacterium*, and *Raoultibacter*, were less abundant. Most core taxa belonged to phyla Firmicutes, Bacteroidota, and Proteobacteria. Notably, Patescibacteria and Verrucomicrobiota were absent in the hindgut core but shared by fecal cores. Meanwhile, Elusimicrobiota was shared exclusively by fecal cores of *B. orientalis* and *P. americana*. The hindgut core of *B. germanica* shared the most taxa with other cores but had the fewest. Moreover, among fecal cores, *B. germanica* had the lowest number of taxa and was the only one without exclusive taxa. The fecal core of *B. orientalis* contained exclusive taxa, including the phylum Cyanobacteria and genera such as *Anaerofustis*, *Sedimentibacter*, *Tuzzerella*, and *Ralstonia*. Finally, the fecal core of *P. americana* had the highest number of total and exclusive taxa, including phyla Synergistota, Fibrobacterota, Campilobacterota, and Halobacterota, and genera such as *Fretibacterium*, *Sulfurospirillum*, *Akkermansia*, *Desulfobotulus*, or the Archaea *Methanimicrococcus*.

## 4. Discussion

In this work, we conducted four experiments, each aimed at answering a specific question. The design has also enabled us to analyze the data more holistically and to understand the factors driving the acquisition of hindgut microbiota in cockroaches. Thus, we analyzed the microbiota of the hindgut and feces from *B. germanica* (our model study) and from *P. americana* and *B. orientalis*. Fecal samples were included because coprophagy plays a role in shaping gut microbiota in gregarious insects [19,47]. The most abundant phyla were Bacteroidota, followed by Firmicutes and Proteobacteria, in concordance with previous studies in *B. germanica* [10,19,20,21,22], *B. orientalis* [48], and *P. americana* [22,23,49,50].

In the first experiment, we planned to compare the microbiota of two genetically identical populations of *B. germanica* separated two decades ago and maintained since then in two different environments (i.e., the rearing place with its associated diet, rB and rV1). We found that both hindgut and fecal samples were more similar within the same environment, indicating that the microbiota in feces reflects that of the hindgut. However, although differences were observed, most taxa were present in all four groups. This suggests that, despite two decades of separation, the transmission of the microbiota through parental coprophagy and the shared genetic background of both populations, which likely promote similar taxon selection, have maintained highly similar microbiotas. These findings further support the host’s essential role in selecting taxa from parental feces. At the same time, our results highlight the importance of environmental factors, including diet and other uncontrolled variables, consistent with previous studies showing that diet is a key determinant of microbiota composition in insects and other animals [15,51,52,53]. They also suggest that the microbiota transmitted over two decades through coprophagy has been subtly shaped by bacteria from multiple environmental sources that were differentially incorporated into the microbiota of the two twinned populations.

Based on these results, a second experiment was designed to examine the effect of the environment (excluding diet) on the microbiota. Two populations were founded from germ-free *B. germanica* individuals reared in place rV1 and subsequently maintained in two different non-sterile environments (rearing places rV1 and rV2 with the same diet), thereby preventing contact with parental feces and avoiding coprophagy. We compared the hindgut microbiota composition from both populations with that of a control population reared in rV1. The lowest diversity and richness were observed in the hindgut of coprophagy-blocked cockroaches (Bge_H_GF_rV1 and Bge_H_GF_rV2), consistent with expectations, as these cockroaches could acquire their microbiota only from the environment (diet and the rearing place). We also found that the microbiota of the two coprophagy-blocked populations were more similar to each other than those of the two populations of cockroaches reared in the same environment and fed the same diet (Bge_H_GF_rV1 and Bge_H_rV1), differing only in the presence or absence of parental fecal inputs. This result highlights the importance of coprophagy as a key strategy for microbiota acquisition in adult cockroaches, as previously shown in some invertebrates [54,55], and to ensure adequate diversity and a stable community structure.

Given that coprophagy is important for hindgut microbiota formation, in the third experiment, we studied the fecal microbiota of three cockroach species to determine and compare their composition. We found that the main factor separating samples is the host species, even when the diet and rearing place are the same, in agreement with previous studies on different laboratory-raised species of the genus *Periplaneta* over several generations under the same conditions [49]. Interestingly, fecal microbiotas from *P. americana* and *B. orientalis*, both members of the family Blattidae, cluster more closely together than those of *B. germanica*, a member of the family Ectobiidae, indicating that host genetics favor certain taxa over others in the gut microbiota. As in previous works [22,56,57,58], the microbiota clustered strongly by host species, indicating a significant relationship between host species identity and gut microbiome composition.

These results from the third experiment indicated that *P. americana* harbored the fecal microbiota most divergent from that of *B. germanica*. Accordingly, *P. americana* was selected as the donor species for the fourth experiment to provide the strongest contrast for assessing host-driven filtering during transplantation. Thus, to elucidate the role of the recipient host species in fecal transplantation, we used germ-free nymphs of *B. germanica* populations reared in two environments (rV1 and rV2) and provided with feces from two donors (*B. germanica* and *P. americana*). Inter-species transplanted hindguts with *P. americana* feces showed lower richness and diversity than intra-species transplanted ones. In other studies, the gut microbiota of *P. americana* showed Shannon diversity values over 5 [50]. In contrast, in this study, *B. germanica* individuals transplanted with *P. americana* feces showed values around 3–4. Taken together, this suggests that the host may select certain taxa within the donor-derived microbiota and that *B. germanica* retains only a subset of the *P. americana* microbiota, consistent with host-driven filtering and limited establishment of non-native taxa. Similar host-driven selection has been described in *Drosophila melanogaster*, which uses distinct genes and mechanisms to protect itself against potentially harmful gut microorganisms [3,59,60]. Comparable effects have also been reported in hemimetabolous insects such as termites and other Blattodea, where gut structure and host characteristics shape microbial assembly [16,61].

Insects rely on several host-driven mechanisms to filter and stabilize their gut microbial communities. Immune-based screening mediated by the Toll, IMD, and JAK/STAT pathways plays a central role in recognizing and restricting non-compatible microorganisms, thereby shaping which taxa can successfully colonize the gut environment [62]. In addition, the micro-environment of the digestive tract, with variations in gut pH, spatial oxygen gradients, and nutrient availability, may act as an ecological filter that favors specific bacterial lineages, as demonstrated in mechanistic studies of insect gut colonization [4]. These processes are consistent with the idea that immune regulation, physiological conditions, and molecular compatibility contribute to shaping how hosts selectively assemble and maintain their microbiota [3]. Although these mechanisms are best understood in *D. melanogaster*, comparable pathways have not yet been examined in cockroaches, and their role in Blattodea remains unclear. Our results are consistent with this view: inter-species transplants showed lower diversity and distinct community structure, and the coprophagy-blocked groups became more similar to each other across environments, indicating that *B. germanica* retains only part of the microbiota it receives. In this context, possible mechanisms in cockroaches may include immune screening, physicochemical constraints of the hindgut, and metabolic compatibility between the host and specific bacterial groups, as suggested for other insects.

Furthermore, we observed greater divergence among the microbiota from feces than among the recipient hindgut samples, across different fecal inputs and environments (different rearing places with the same diet), indicating that the host species primarily influences microbiota composition. This suggests that the host species may play a more prominent role than the other studied factors and reinforces the idea that the host shapes its gut microbiota [3]. Nevertheless, the effect of the species donor cannot be ruled out, as can be seen in the PCA (Figure 6), because the hindgut microbiota of transplanted *B. germanica* receiving *P. americana* feces separated from the hindgut microbiota of control cockroaches and hindguts transplanted with *B. germanica* feces. However, the specific host mechanisms underlying this filtering in *B. germanica* remain unknown. Based on other insect systems, a combination of immune regulation and hindgut physiology may determine which taxa can successfully colonize the community. Testing this would require experimental approaches that integrate microbiome manipulation with host physiological responses.

Likewise, the fecal core microbiota of three ubiquitous cockroach species were characterized comparatively. This is of particular interest as these cockroaches are common pests associated with pathogen dissemination, antimicrobial resistance, and allergen production [63,64], all of which represent important public health concerns. Our finding that gut microbiota assembly is strongly shaped by host species identity, fecal inputs, and environmental conditions suggests that factors influencing microbial acquisition and maintenance may indirectly affect microbiome-associated traits relevant to health risks in urban settings. Although pathogen transmission and allergen production were not directly addressed here, this work provides a useful framework for future studies exploring microbiome-informed approaches to cockroach control and risk mitigation within a One Health framework.

A key limitation of this study concerns not only sample size but also the replication structure. In all experiments, individuals within each treatment came from the same colony. Hence, the colony is the experimental unit, and the samples should be treated as subsamples rather than fully independent replicates. This means we may not fully capture variation across colonies, and some of the observed differences could reflect colony-specific effects. In addition, the limited sample size per group may reduce the ability to detect more subtle differences in microbiota composition. For these reasons, the results should be interpreted as patterns observed under controlled experimental conditions, rather than as directly generalizable to broader populations. Despite this, consistent trends were observed across the four experimental designs, suggesting that the main patterns identified are robust within the system studied. Future work, including independent colony replicates and larger sample sizes, will help determine how broadly these results apply.

## 5. Conclusions

Taken together, the results of the four experiments lead us to conclude that the acquisition of the gut microbiota in adult cockroaches is a multifactorial process. Therefore, different aspects of the environment (e.g., diet and rearing place) play a role in the development of their mature microbiota [65], with feces being crucial for establishing and developing the cockroach gut microbiota. However, our results suggest that the host species is the main factor shaping the gut microbiota under the conditions tested. Future work should test which host factors are most important (i.e., immune filtering versus hindgut physicochemical constraints) and how they interact with microbe–microbe interactions during colonization to better understand gut microbiota acquisition in adult insects.

## Data Availability

Sequence data were deposited in the European Nucleotide Archive under study accession PRJEB85908. Samples’ metadata are included in Supplementary Table S1. Data and code used for this work are available on GitHub “https://github.com/tbcgit/Blattella_study” (accessed on 9 April 2025).

## Supplementary Materials

The following supporting information can be downloaded at: https://www.mdpi.com/article/10.3390/insects17060615/s1, Figure S1: Microbiome composition barplots. The most abundant taxa are shown, representing the top 10 phyla (A) and top 30 genera (B) in terms of relative abundance. The remaining taxa are summarized under the “other” taxon. Background colors and identifying labels match those used to name the experiments in Figure 1; Figure S2: Interactive PCA plot with CLR-transformed data at the ASV level for the first three principal components, PC1, PC2, and PC3. Related to Figure 2C; Figure S3: Interactive PCA plot with CLR-transformed data at the genus level for the first three principal components, PC1, PC2, and PC3. Related to Figure 2D; Figure S4: Heatmap of the differentially abundant genera in the fecal transplant design. Z-scored average relative abundances by group are shown for the 93 significant taxa identified by ALDEx2. On the left side, Log10 (average relative abundances) by group are also displayed, where gray cells represent absent taxa in the group; Figure S5: Core microbiome analysis. Taxonomic relationships between different core genera for the hindgut of *B. germanica*, including rV1 and rB (A), feces of *B. germanica* including rB, rV1, and rV2 (B), feces of *B. orientalis* in rV2 (C), and feces of *P. americana* in rV2 (D). Only genera detected in all the samples of each grouping in standard conditions were considered with a minimum relative abundance threshold of 0.0001. Color axes represent the core uniqueness of the different taxonomic levels, whereas the terminal nodes represent the Log10 (average relative abundance) by grouping of the different core genera; Table S1: Detailed metadata of cockroach groups and samples; Table S2: Alpha diversity tests at the ASV level based on Observed Richness and Shannon’s Diversity Index. For each metric, the results of the *t* tests are reported, along with the Shapiro and Levene tests used to verify the assumptions for their applicability. Adjusted *p*-values were corrected with the BH method. A significance level threshold of 0.05 was set; Table S3: Global beta diversity tests at ASV level based on Aitchison distances. All groups were compared using a global and pairwise PERMANOVA. A model using the “betadisper” function was also computed to test for homogeneity across global and pairwise groups using the permutest and TukeyHSD, respectively. Adjusted *p*-values were corrected with the BH method. A significance level threshold of 0.05 was set; Table S4: Global beta diversity tests at genus level based on Aitchison distances. All groups were compared using a global and pairwise PERMANOVA. A model using the “betadisper” function was also computed to test for homogeneity across global and pairwise groups using the permutest and TukeyHSD, respectively. Adjusted *p*-values were corrected with the BH method. A significance level threshold of 0.05 was set; Table S5: Particular beta diversity PERMANOVA tests at the genus level for the different experimental designs based on Aitchison distances. A significance level threshold of 0.05 was set. Results are separated by experimental analyses (A1–A4); Table S6: Presence-absence data at the genus level. Related to Venn diagrams and upset plots. Taxa were considered to be present in the groups if detected in at least one sample. Results are presented globally (All) and separated by experimental analyses (A1–A4); Table S7: ALDEx2 difference abundance results at the genus level for the fecal transplant design. GLM and KW tests for one-way ANOVA with two or more conditions were used. All *p*-values were adjusted using the BH method. A significance level threshold of 0.05 was set; Table S8: Presence–absence data for the core genera. Only genera detected in all samples of each grouping in standard conditions were considered with a minimum relative abundance threshold of 0.0001.

## Abbreviations

The following abbreviations are used in this manuscript:

16S rRNA	16S ribosomal RNA gene
ANOVA	Analysis of Variance
ASV	Amplicon Sequence Variant
BCN	Barcelona
Bge	*Blattella germanica*
BgeF	Fecal input from *B. germanica* during transplantation
BH	Benjamini–Hochberg
BHI	Brain Heart Infusion
Bor	*Blatta orientalis*
CLR	Centered Log-Ratio transformation
F	Feces
GF	Germ-Free
GLM	Generalized Linear Model
H	Hindgut
I2SysBio	Institute for Integrative Systems Biology
IBE	Institute of Evolutionary Biology
ICBiBE	Cavanilles Institute of Biodiversity and Evolutionary Biology
IMD	Immune Deficiency
JAK/STAT	Janus Kinase/Signal Transducers and Activators of Transcription
KW	Kruskal–Wallis
ns	Not Significant
Pam	*Periplaneta americana*
PamF	Fecal input from *P. americana* during transplantation
PCA	Principal Component Analysis
PERMANOVA	Permutational Multivariate Analysis of Variance
rB	rearing place Barcelona (IBE)
rV1	rearing place Valencia1 (I2SysBio)
rV2	rearing place Valencia2 (ICBiBE)
SDS	Sodium Dodecyl Sulfate
USA	United States of America
VLC	Valencia
