# Supplementary figures and images for: Blattella germanica Selects Microbiota Taxa from Feces and Environmental Inputs
Source: Insects. 2026 Jun 10;17(6):615. doi: 10.3390/insects17060615 (PMC13300209; doi:10.3390/insects17060615)

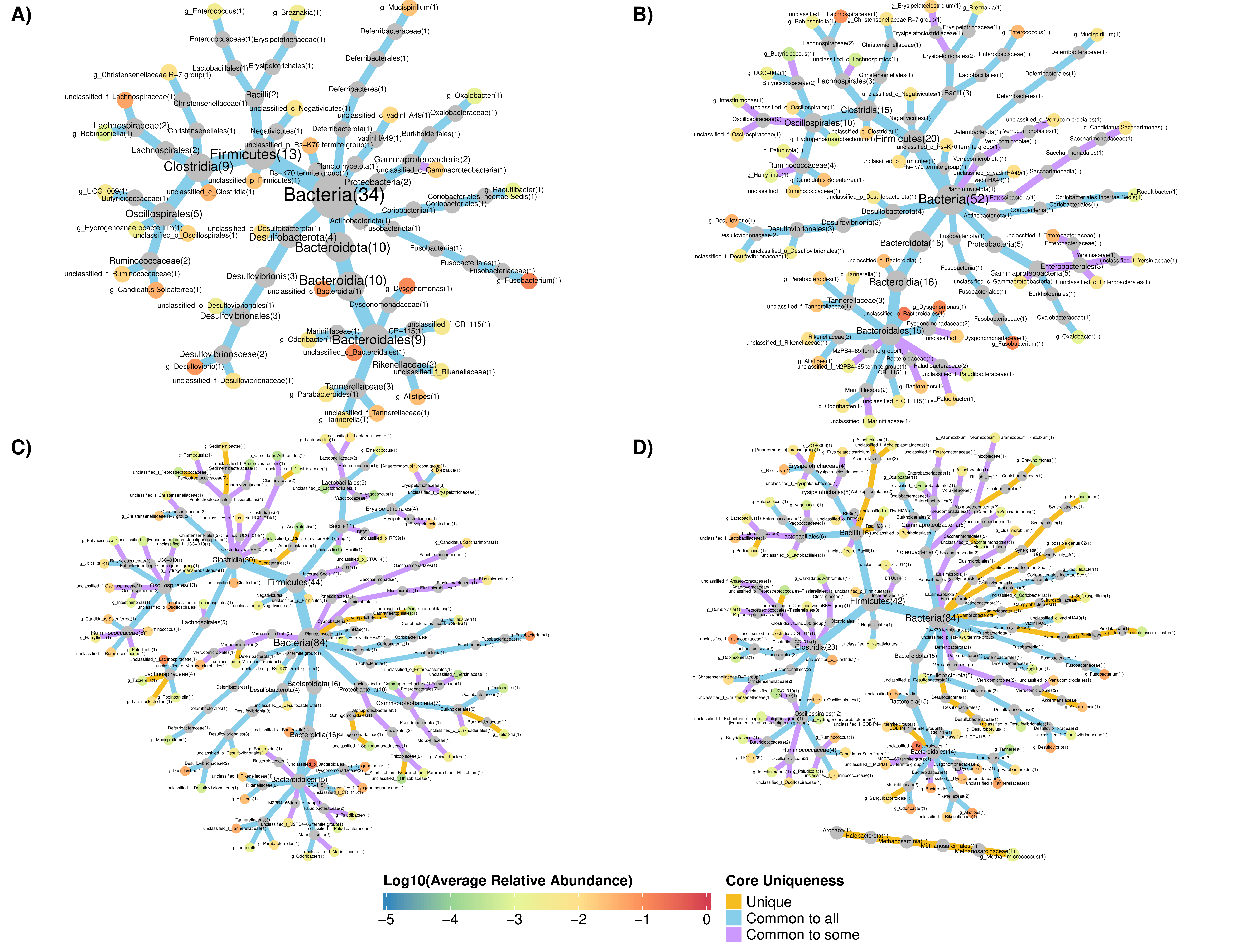

Supplement: Supplementary file 1 [file insects-17-00615-s001.zip › Supplementary_FigureS5_convert600dpi.png]
